# Load estimation data using direct search method based on pattern search

**DOI:** 10.1016/j.dib.2024.110797

**Published:** 2024-08-03

**Authors:** Leonardo Ramos Pereira, Giovanni Manassero

**Affiliations:** a APROT Engenharia. Av. Engenheiro Luís Carlos Berrini, 550 - Itaim Bibi, São Paulo SP, 04571-020, Brazil; b Escola Politécnica da Universidade de São Paulo. Av. Prof. Luciano Gualberto, Travessa 3, 158, Butantã, São Paulo SP, 05508-010, Brazil

**Keywords:** Constrained optimization, Distribution system, Mathematical optimization, Power system

## Abstract

Accurately estimating load is essential for effective electric distribution planning, assets management, precise power flow predictions, accurate power losses calculations, and efficient integration of distributed energy resources. This work describes a dataset that was generated using Matlab and OpenDSS to produce several simulations in which load estimation is performed using a direct search method called pattern search. These simulations were conducted on three typical distribution feeders (IEEE 13-bus, 37-bus, and 123-bus) that support studies in distribution planning, assets management, power flow predictions, power losses calculations, and distributed resource integration. The dataset includes individual demand profiles of residential, commercial, and industrial consumers specified for the three distribution feeders, comprising 96 distinct scenarios. An optimization method was developed to obtain the dataset, which employs the pattern search technique to estimate loads through the optimization of objective functions and specified constraints. The load estimation quality was assessed for all three feeders, utilizing estimation quality indices proposed by the authors. These indices evaluated both the initial and proposed load estimation methods across the developed scenarios. Furthermore, the data provided in this article can be utilized for comparison with future load estimation studies, particularly regarding the quality of the method's results.

Specifications Table

**Table:** 

Subject	*Electrical and Electronic Engineering*
Specific subject area	*Load Estimation on Distribution Power Systems*
Data format	Raw, Analyzed, Filtered
Type of data	Table, Image, Chart, Graph, Figure
Data collection	*For three theoretical circuit models made available by OpenDSS (IEEE 13, 37 and 123 bars), an algorithm was run in Matlab that sought to estimate the load of these circuits based on knowledge of voltage and current values at the voltage source and at specific points in the circuit. The estimation was carried out using a direct search method that uses the pattern search method. In addition to measurements in the circuit, voltage restrictions, power factor and transformer loading were used.*
Data source location	*Universidade de São Paulo* *Escola Politécnica* *Departamento de Engenharia de Energia e Automação Elétricas* *Av. Prof. Luciano Gualberto, Travessa 3, 158* *CEP: 05508–010 – São Paulo – SP - Brazil*
Data accessibility	Repository name: Ramos Pereira, Leonardo (2024), “The performance of the pattern search direct search method in solving load estimation problems”, Mendeley Data, V1 [2] Data identification number: 10.17632/mr79876xr2.1 Direct URL to data: 10.17632/mr79876xr2.1 Instructions for accessing these data: The data is available in the public domain
Related research article	*Ramos Pereira, Leonardo, and Giovanni Manassero. “Performance of the pattern search direct search method for load estimation in distribution grids”. 2024 IEEE PES Innovative Smart Grid Technologies Europe (ISGT Europe). IEEE, 2024.*

## 1 Value of the Data

Key Points:

• The data offers insights into load estimation methodologies for IEEE circuits with varying load complexities.
• It provides a detailed exploration of 96 simulation scenarios, allowing researchers to understand the impact of formulation variations on load estimation.
• Researchers can leverage the results to propose new evaluation indices, compare with alternative estimation methods, and reference outcomes detailed in related studies.

## 2 Background

In an electrical system, distributors do not have information about the magnitude of loads connected to the system. A system that provides this estimation in real time would allow better management of electrical system assets. The related article proposes a method for this estimation and tests this method using three theoretical circuits.

The method uses the relationship between the magnitude of the loads and the voltages and currents measured at the feeder to estimate the loads using a direct search method.

The quality of the estimation from the method can be improved based on imposed restrictions, such as a power factor greater than 0.5 or transformer loading less than 1.25 pu. These restrictions are tested for the three circuits and their influence on the results is analyzed.

The estimation must start from a suggested initial value for the magnitude of the loads. This initial value is generated from a method that divides the power measured in the feeders proportionally to the nominal power of each load in relation to the total nominal power. This method is also evaluated.

## 3 Data Description

**Fig. 1:**
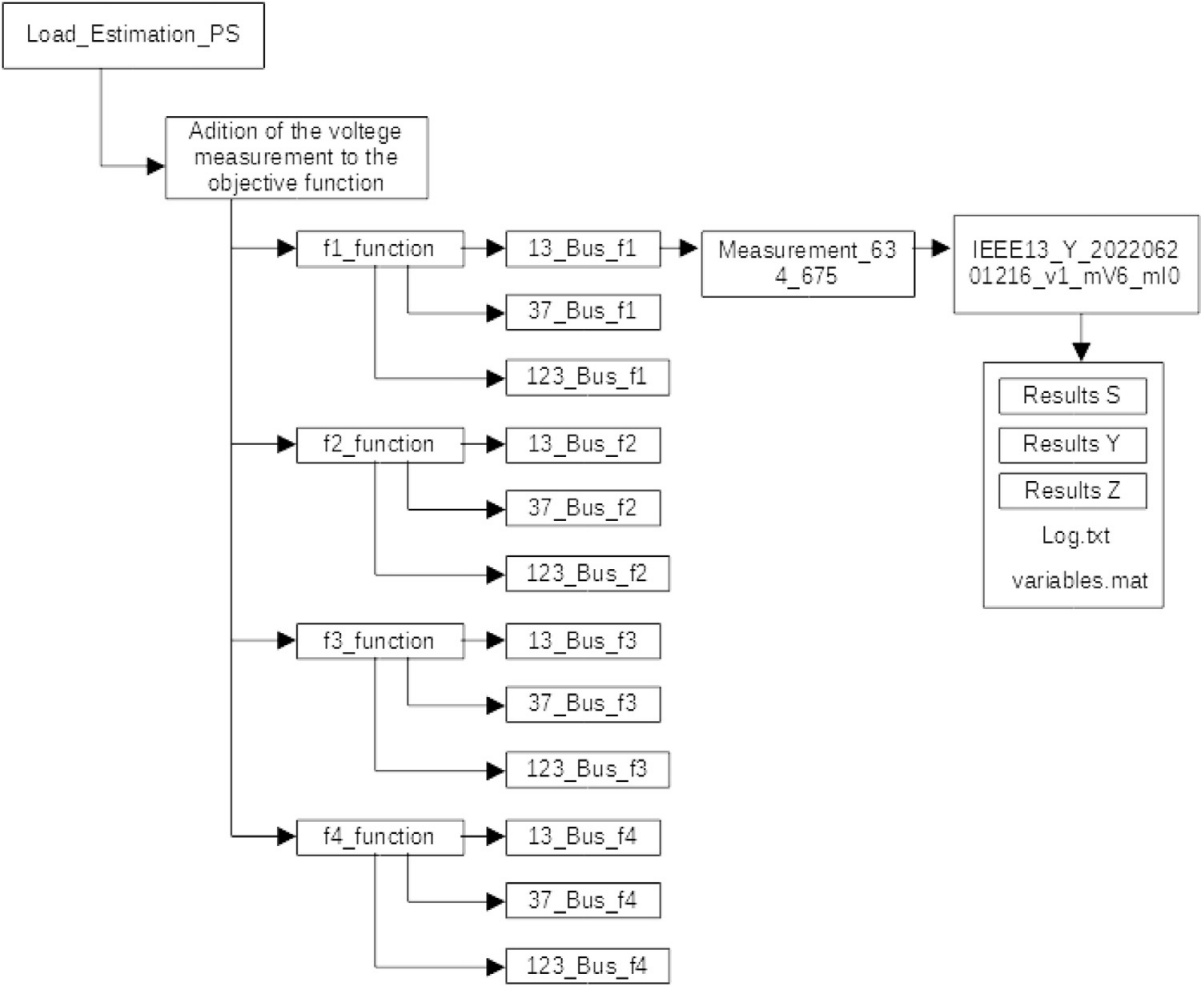
Dataset folder tree structure example.

The dataset corresponding to the estimation results is organized by folder and subfolder level, and the meaning of each level is illustrated in Table 1 and Fig. 1.

**Table 1:** “Dataset Folder structure”.

Level	Description	Folder Name
0	Load_Estimation_PS folder	Load_Estimation_PS
1	Simulation Condition	Impact of the Initial Estimate
		Unconstrained objective Functions
		Power Factor Constraint
		Voltage Level Constraint
		Constraint based on transformer loading
		Constraint based on voltage measurement
		Constraint based on current measurement
		Addition of voltage measurement to the objective function
		Addition of current measurement to the objective function
2	Objective Function Evaluated	f1_Function
		f2_Function
		f3_Function
		f4_Function
3	Feeder simulated	13_Bus_fx^1^ (IEEE 13 Bus)
		37_Bus_fx (IEEE 37 Bus)
		123_Bus_fx (IEEE 123 Bus)
4	Identification of bars with voltage measurement or elements with current measurement (if any)	*See figure 1 example.*
5	Simulation ID	*See figure 1 example. As detailed below.*
6	Simulation files and folders	“Results S” folder; “Results Y” folder; “Results Z” folder; “Log.txt” file; “variables.mat” file
7	“Results S” folder	“FigS.png” file; “ResumeS.csv” file; “Scomp1.csv” file; “TabFreqS.csv” file;
7	“Results Y” folder	“FigY.png” file; “ResumeY.csv” file; “Scomp1.csv” file; “TabFreqY.csv” file;
7	“Results Z” folder	“FigZ.png” file; “ResumeZ.csv” file; “Scomp1.csv” file; “TabFreqZ.csv” file;

1 x is the objective function evaluated in the feeder.

The simulation ID is a folder whose name has the format:

IEEE<busnumber>_Y_<Date>_<version>_mV<vmeasure>_mI<imeasure><constraints indicator>, where:

• <busnumber>: number of buses in the circuit (13, 37 or 123)
• <Date>: sequence of numbers that represents the simulation time in the format <Year><Month><Day><Hour><Minute>.
• <version>: formulation of the objective function considered in the simulation (v1, v2, v3 or v4);
• <vmeasure>: number of bars with voltage measurement
• <imeasure>: number of bars with current measurement
• <constrains indicator>: if there are restrictions, it takes the value "_NC". If this is not the case, this field does not appear in the folder name

The simulation files are:

• Simulation results expressed by physical quantity, saved in the ResultsS, ResultsZ and ResultsY folders;
• “Log.txt”contain simulation parameters, logs and results in text format;
• “variables.mat”: variables from simulation exported from Matlab, for later processing without the need to run the simulation. The exported variables are described in Table 2;

**Table 2:** “variables.mat” variables exported from each simulation.

Variable Name	Description	Data Type
dados	Data from simulated circuit	Struct
dados.barraVmed	Bars with voltage measurement	Cell array;
dados.elemImed	Element’ list with current measurement	Cell array;
dados.barras	Number of buses in circuit	Double;
dados.name	Circuit name	String;
dados.escolhadiv	equals to 1 if the circuit is divided by measure points; equals to −1, otherwise	double;
dados.subredes	if dados.escolhadiv = 1, this list contains buses list separated by sub-circuit	Cell array;
dados.node_order	node list of the circuit	Cell array;
dados.ptos_medV	number of elements in dados.barraVmed;	double;
dados.ptos_medI	number of elements in dados.elemImed;	double;
dados.ptosmedSource;	number of phases with measurement in the feeder of circuit;	double
dados.Ysistema.list	Admitance matrix of the circuit in a table, ordinated according dados.node_order	Cell array;
dados.Yrede.list	Admitance matrix of the circuit without loads in a table, ordinated according dados.node_order	Cell array;
dados.Yload.list	Admitance matrix of the circuit loads in a table, ordinated according dados.node_order	Cell array;
dados.YLoadError	variable to error check in dados.Yload generation	Cell array;
dados.Vorder	List of nodal voltages, ordinated according dados.node_order	Cell array;
dados.Iorder	List of current nodal injections, ordinaded according dados.node_order	Cell array;
dados.Load	Circuit's Load characterisitics list	Cell array;
dados.Yraiz	True value of the load admitances in the circuit, to validate the estimation	Cell array;
dados.Zraiz	True value of the load impedances, to validate the estimation	Cell array;
dados.Sraiz	True value of the load powers, to validate the estimation	Cell array;
dados.Ypos	position of the load's on admittance matrix	Cell array;
dados.medI_list	Extended element’ list with current measurement.	Cell array;
dados.trafoList	Circuit's power transformers list	Cell array;
mainset	Simulation configurations	Cell array;
mainset.const	precision multiplier for search	double;
mainset.dom	Search domain (equals to 1 for admittance);	double;
mainset.version	Objective function evaluated (1 for f1, 2 for f2, 3 for f3 and 4 for f4);	double;
mainset.restriction	Equals to 0 if there aren't restrictions; 1, otherwise	double;
mainset.method	equals to 0 to utilize pattern search matlab's tool	double;
mainset.time	data when simulation was executed	double array;
mainset.string_dom	String search domain (‘Y’ for impedance)	string;
options	matlab pattern search tool options (see documentations [4] for details)	struct
search	search parameters related to the circuit	struct
search.Yraiz	True value of the load admitances in the circuit, to validate the estimation	double array;
search.Zraiz	True value of the load impedances in the circuit, to validate the estimation	double array;
search.Sraiz	True value of the load power in the circuit, to validate the estimation	double array;
search.Raiz	True value of the load admitances in the circuit, to validate the estimation	double array;
search.PlotFunctions	Functions to plot estimations from pattern search from each iteraction	double array;
search.chute_inicial	Initial estimative used by pattern search method	double array;
search.UB	Superior boundarie for search	double array;
search.LB	Inferior boundarie for search	double array;
masRes	list with the maximum and minimum errors, mean and standard deviation of the estimate, expressed in absolute and percentage forms	cell array;
resultsV	set of lists containing the bus voltage estimation errors	struct
resultsV.ErroV1	For each bus: $ErroV1=\frac{|V_{est}\left|-\right|V_{true}|}{|V_{est}|}$^2^	double array
resultsV.ErroV2	For each bus: $ErroV2=\frac{|V_{est}\left|-\right|V_{true}|}{|V_{est}|}$	double array
resultsV.ErroV3	For each bus: ErroV3=$\frac{Re\left(V_{est}\right)-Re\left(V_{true}\right)}{Re(V_{est})}$	double array
resultsV.ErroV4	For each bus: : ErroV4=$\frac{Im\left(V_{est}\right)-Im\left(V_{true}\right)}{Im(V_{est})}$	double array
resultsV.ErroV	table with ErroV1, ErroV2, ErroV3 and ErroV4 for comparison	cell array
resultsS	description of results in which the load is expressed by its power	struct
resultsS.resume	list with actual load values, estimated values and calculated errors for each bar; this list is written in the “ResumeS.csv” file explained below	struct
resultsS.id	physical quantity used to describe the estimation results, 'S' is used	string
resultsS.chute_inicial	identical to search.chute_inicial, but in list format	cell array
resultsS.Estimado	Estimated load	cell array
resultsS.maxResume	identical to maxRes	cell array
resultsS.tabFreq	Frequency table for erros calculated in resultsS.resume; this table is written in the “TabFreq.csv” file explained below	cell array
resultsS.resumeinic	contains the same fields present in resultsS.resume calculated for the resultsS.chute_inicial estimate	cell array
resultsS.maxResumeinic	contains the same fields present in resultsS.maxResume calculated for the resultsS.chute_inicial estimate	cell array
resultsS.tabFreqinic	contains the same fields present in resultsS.tabFreq calculated for the resultsS.chute_inicial estimative	cell array
resultsS.compare1	difference between resultsS.resume and resultsS.resumeinic; this list is written in the “Scomp1.csv” file explained below	cell array
results.compare2	diferente between resultsS.maxResume and resultsS.maxResumeinic	cell array
hist	information for constructing the histogram from the frequency table	struct
resultsY	description of results in which the load is expressed by its admittance (the attributes of this structured variable are analogous to the attributes of resultsS)	struct
resultsZ	description of results in which the load is expressed by its impedance (the attributes of this structured variable are analogous to the attributes of resultsS)	struct

2 *V*_*est*_ is estimated value for each bus voltage; *V*_*true*_ is true value; Re(*x*) is real part from complex number *x*; Im(*x*) is imaginary part from complex number *x*.
  The files relevant to the ‘Results S’ folder will be described, considering that the files in the ‘ResultsY’ folder and the ‘ResultsZ’ folder are analogous. These files are:

•ResumeS.csv - result of power estimation for each load and associated errors.^1^ An example is showed on Table 3;

**Table 3:** “Results for IEEE 13 bus circuit” example.

Var1	Var2	Var3	Var4	Var5	Var6	Var7	Var8	Var9
**611.3–611**	158.95	74.8	157.05	75.73	1.2	−2.15	1.19	−1.24
**634.1–634a**	156.58	107.65	165.65	79.87	15.38	25.4	−5.79	25.81
**634.2–634b**	121.47	91.1	133.03	64.14	19.32	30.18	−9.51	29.59
**634.3–634c**	116.79	87.6	129.17	62.28	19.3	30.18	−10.6	28.9
**645.2–645**	176.12	129.5	190.74	91.97	18.42	29.14	−8.31	28.98
**646.2.3–646**	234.33	134.48	236.6	114.08	7.6	13.77	−0.97	15.17
**652.1–652**	122.37	82.21	128.13	61.78	14.4	24.06	−4.71	24.85
**670.1–670a**	17.21	10.12	17.31	8.35	8.91	15.5	−0.59	17.54
**670.2–670b**	69.98	40.29	70.36	33.93	7.89	14	−0.55	15.79
**670.3–670c**	115.65	67.22	118.33	57.06	7.86	14.66	−2.31	15.12
**671.1.2.3–671.1.2**	382.98	218.84	385.85	186.05	7.46	13.46	−0.75	14.99
**671.1.2.3–671.2.3**	392.93	224.53	399.47	192.62	7.2	13.46	−1.67	14.21
**671.1.2.3–671.3.1**	372.07	212.61	375.51	181.06	7.41	13.46	−0.92	14.84
**675.1–675a**	464.97	182.15	434.19	209.36	8.23	−20.33	6.62	−14.94
**675.2–675b**	73.45	64.81	85.32	41.14	27.03	37.86	−16.15	36.52
**675.3–675c**	272.36	199.1	301.7	145.47	18.12	28.83	−10.77	26.94
**692.3.1–692**	164.29	145.93	192.55	92.84	27.37	38.14	−17.2	36.38
Where:

­ Var1: name of the bus
­ Var2: Real part of the load's true power, in the CSV file the column name is Re(Sraiz);
­ Var3: Imaginary part of the load's true power;
­ Var4: Real part of the estimated load power;
­ Var5: Imaginary part of the estimated load power;
­ Var6: percentage of the modulus of the difference between the true and estimated powers in relation to the true power.
  $Erro\;Abs\left(Delta\right)\left(\%\right)=100.\frac{|S_{raiz}-S_{est.}|}{|S_{raiz}|}$
­ Var7: percentage of the difference between the arguments in relation to the true argument.
  $Erro\;Delta\left(Arg\right)\left(\%\right)=100.\frac{\arg\left(S_{raiz}\right)-\arg\left(S_{est}\right)}{\arg(S_{raiz})}$
­ Var8: Percentage estimation error in relation to the real part in relation to the true nominal active power.
  $Erro\;Real\left(\%\right)=100.\frac{Re\left(S_{raiz}\right)-Re\left(S_{est}\right)}{Re(S_{raiz})}$
­ Var9: Percentage estimation error in relation to the imaginary part in relation to the true nominal reactive power.
  $Erro\;Imag\left(\%\right)=100.\frac{Im\left(S_{raiz}\right)-Im\left(S_{est}\right)}{Im(S_{raiz})}$

• TabFreqS.csv - table of percentage error frequencies acumulated, based on the “ResumeS.csv” table considering error in module, in phase, error in the real part and error in the imaginary part^2^; Table 4 presents an example.

**Table 4:** Frequency table for percentage errors in the results table S.

Var1	Var2	Var3	Var4	Var5
**0**	0	0	0	0
**0.1**	0	5.88	5.88	5.88
**0.2**	0	5.88	11.76	5.88
**0.3**	0	5.88	11.76	5.88
**0.4**	0	5.88	23.53	5.88
**0.5**	0	5.88	35.29	5.88
**0.6**	0	5.88	41.18	5.88
**0.7**	0	11.76	41.18	5.88
**0.8**	0	11.76	41.18	5.88
**0.9**	0	11.76	41.18	5.88
**1**	0	11.76	41.18	5.88
**2**	0	11.76	41.18	5.88
**3**	5.88	17.65	41.18	5.88
**4**	5.88	17.65	52.94	5.88
**5**	5.88	23.53	52.94	5.88
**6**	5.88	23.53	52.94	5.88
**7**	11.76	23.53	52.94	11.76
**8**	35.29	23.53	70.59	11.76
**9**	58.82	23.53	70.59	11.76
**10**	64.71	23.53	70.59	11.76
**20**	76.47	70.59	88.24	70.59
**30**	94.12	82.35	94.12	88.24
**40**	100	94.12	100	100
**50**	100	100	100	100
**60**	100	100	100	100
**70**	100	100	100	100
**80**	100	100	100	100
**90**	100	100	100	100
**100**	100	100	100	100
**Inf**	100	100	100	100

Where:

­ Var1: Percentage error range
­ Var2: Percentage of loads whose calculated error from the difference between the module of the estimated power and the real power (column var6 in the table ResumeS.csv) does not exceed the value of the corresponding percentage error range.
­ Var3: Percentage of loads whose calculated error from the difference between the argument of the estimated power and the real power (column var7 in the table ResumeS.csv) does not exceed the value of the corresponding percentage error range.
­ Var4: Percentage of loads whose calculated error from the difference between the real part of the estimated power and the real power (column var8 in the table ResumeS.csv) does not exceed the value of the corresponding percentage error range.
­ Var5: Percentage of loads whose calculated error from the difference between the imaginary part of the estimated power and the real power (column var9 in the table ResumeS.csv) does not exceed the value of the corresponding percentage error range.

**Fig. 2:**
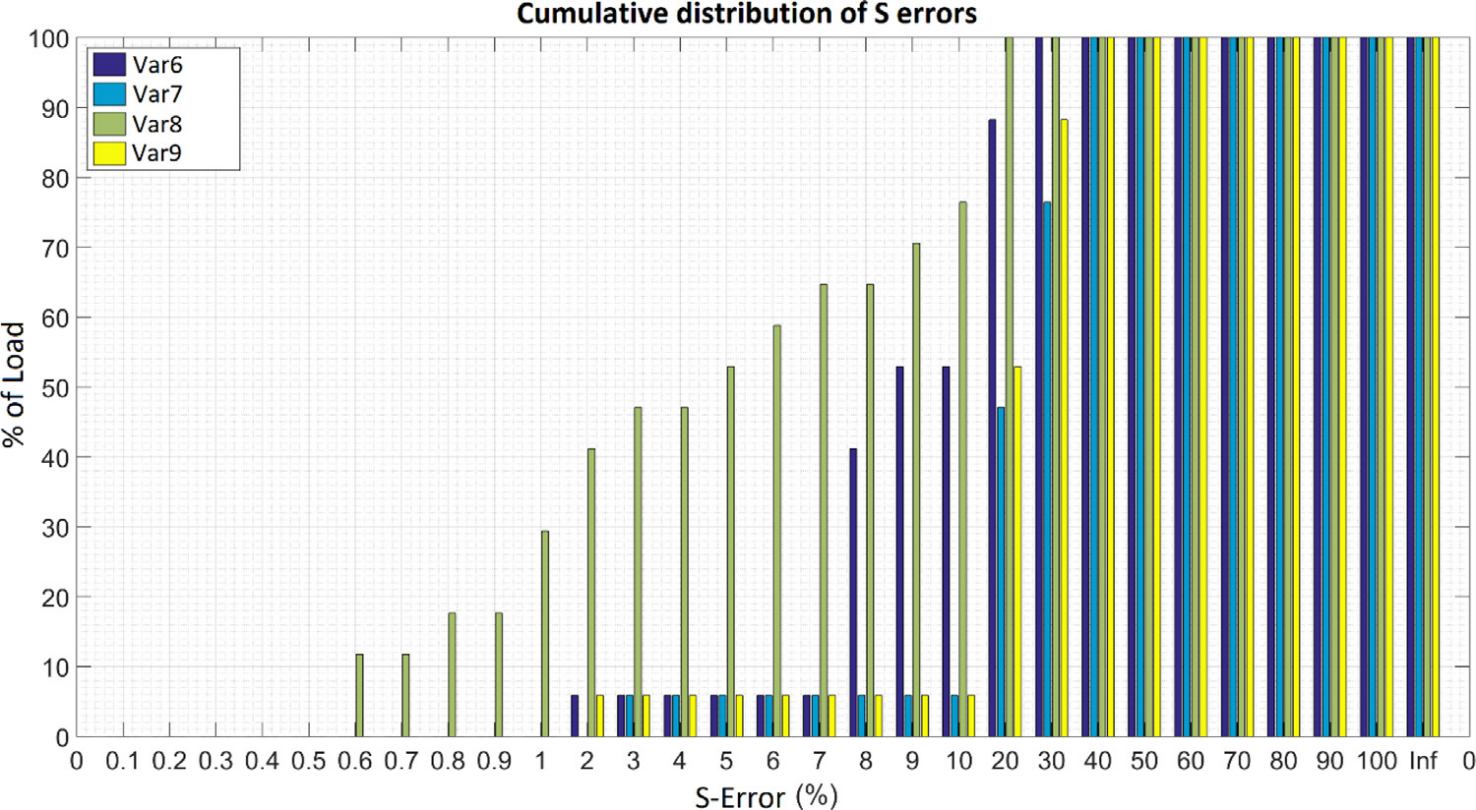
Example of a histogram based on the TabFreq.csv table.

• FigS.png - histogram based on the frequency table “TabFreqS.csv”; Fig. 2 illustrates a histogram.

Where:

­ Var6: percentage of the modulus of the difference between the true and estimated powers in relation to the true power.
  $Erro\;Abs\left(Delta\right)\left(\%\right)=100.\frac{|S_{raiz}-S_{est.}|}{|S_{raiz}|}$
­ Var7: percentage of the difference between the arguments in relation to the true argument.
  $Erro\;Delta\left(Arg\right)\left(\%\right)=100.\frac{\arg\left(S_{raiz}\right)-\arg\left(S_{est}\right)}{\arg(S_{raiz})}$
­ Var8: Percentage estimation error in relation to the real part in relation to the true nominal active power.
  $Erro\;Real\left(\%\right)=100.\frac{Re\left(S_{raiz}\right)-Re\left(S_{est}\right)}{Re(S_{raiz})}$
­ Var9: Percentage estimation error in relation to the imaginary part in relation to the true nominal reactive power.
  $Erro\;Imag\left(\%\right)=100.\frac{Im\left(S_{raiz}\right)-Im\left(S_{est}\right)}{Im(S_{raiz})}$

• Scomp1.csv - table that records the difference between the error modules between the simulation performed and the simulation that considers the initial estimate^3^; Table 5 presents an example.

**Table 5:** Frequency table for percentage errors in the results table S.

Var1	Var2	Var3	Var4	Var5
**611.3–611**	5.21	12.43	2.56	11.55
**634.1–634a**	−1.21	−1.94	4.37	−5.62
**634.2–634b**	1.84	−30.14	11.67	−8.48
**634.3–634c**	11.75	18.64	23.18	−3.4
**645.2–645**	−11.36	−28.48	−0.93	−22.53
**646.2.3–646**	0.49	0	−0.66	1.08
**652.1–652**	−11.76	−21.6	−1.53	−24.83
**670.1–670a**	0.42	0	−0.16	0.84
**670.2–670b**	0.22	0	−0.55	0.46
**670.3–670c**	0.53	−1.94	−1.8	1.54
**671.1.2.3–671.1.2**	−0.12	−1.28	−0.35	−0.2
**671.1.2.3–671.2.3**	0.61	0	−1.52	1.53
**671.1.2.3–671.3.1**	0.52	0	−0.52	1.12
**675.1–675a**	0.62	0	1.32	−1.63
**675.2–675b**	0	0	−0.36	0.19
**675.3–675c**	−7.79	−24.67	−2.96	−13.07
**692.3.1–692**	−0.02	0	−1.54	0.84

Where:

­ Var1: name of the bus
­ Var2: Percentage error difference (calculated from the difference between the module of the estimated power and the real power - column var6 in the table ResumeS.csv) between the estimate obtained with PS and the initial estimate.
­ Var3: Percentage error difference (calculated from the difference between the argument of the estimated power and the real power – column var7 in the table ResumeS.csv) between the estimate obtained with PS and the initial estimate.
­ Var4: Percentage error difference (calculated from the difference between the real part of the estimated power and the real power - column var8 in the table ResumeS.csv) between the estimate obtained with PS and the initial estimate.
­ Var5: Percentage error difference (calculated from the difference between the imaginary part of the estimated power and the real power - column var9 in the table ResumeS.csv) between the estimate obtained with PS and the initial estimate.

In the example above, the power module estimation error for load 611 worsened by approximately 5.21 % in relation to the error related to the initial estimate. The files in the “Results Y” and “Results Z” folders are analogous and contain the same estimates using admittance and impedance, respectively.

## 4 Experimental Design, Materials and Methods

### 4.1 Methodology description

The proposed methodology begins with the admittance matrix of the system without loads, with is supposed to be known:

$$\begin{array}{ccc} \left[Y_{net}\right] & = & \left[\begin{array}{cccccccc} \left[Y_{11}\right] & \left[Y_{12}\right] & \ldots & \left[Y_{1p}\right] & \ldots & \left[Y_{1q}\right] & \ldots & \left[Y_{1n}\right]\\ \left[Y_{21}\right] & \left[Y_{22}\right] & \ldots & \left[Y_{2p}\right] & \ldots & \left[Y_{2q}\right] & \ldots & \left[Y_{2n}\right]\\ \left[Y_{32}\right] & \left[Y_{32}\right] & \ldots & \left[Y_{3p}\right] & \ldots & \left[Y_{3q}\right] & \ldots & \left[Y_{3n}\right]\\ \vdots & \vdots & \ddots & \vdots & \ddots & \vdots & \ddots & \vdots \\ \left[Y_{p1}\right] & \left[Y_{p2}\right] & \ldots & \left[Y_{pp}\right] & \ldots & \left[Y_{pq}\right] & \ldots & \left[Y_{pn}\right]\\ \vdots & \vdots & \ddots & \vdots & \ddots & \vdots & \ddots & \vdots \\ \left[Y_{q1}\right] & \left[Y_{q2}\right] & \ldots & \left[Y_{qp}\right] & \ldots & \left[Y_{qq}\right] & \ldots & \left[Y_{qn}\right]\\ \vdots & \vdots & \ddots & \vdots & \ddots & \vdots & \ddots & \vdots \\ \left[Y_{n1}\right] & \left[Y_{n2}\right] & \ldots & \left[Y_{np}\right] & \ldots & \left[Y_{nq}\right] & \ldots & \left[Y_{nn}\right] \end{array}\right],\\ \text{in}\;\text{wich}\;\left[Y_{ij}\right] & = & {\left[\begin{array}{ccc} {\dot{Y}}_{AA} & {\dot{Y}}_{AB} & {\dot{Y}}_{AC}\\ {\dot{Y}}_{BA} & {\dot{Y}}_{BB} & {\dot{Y}}_{BC}\\ {\dot{Y}}_{CA} & {\dot{Y}}_{CB} & {\dot{Y}}_{CC} \end{array}\right]}_{ij} \end{array}$$

In wich [Yij] represents a nodal admittance matrix by phases A, B and C. It is supposed to be know the nodes in wich loads connect and the position p and q of this loads in [Y_net_]. Thus, being $\left[\bar{y_{pp}}\right]$ and $\left[\bar{y_{qq}}\right]$ the admittance matrices of the loads whose elements are contained in the load vector [y_load_], adding $\left[\bar{y_{pp}}\right]$ and $\left[\bar{y_{qq}}\right]$ with the matrices [Y_pp_] and [Y_qq_], we obtain the admittance matrix of the system with loads [Y_system_] = [Y_system_([y_load_])]. By employing the Kron reduction, a relationship between the voltages and currents in the feeder is obtained, explicitly showing the dependence of this relationship on the admittance of the loads:

$$\left[\begin{array}{c} {\dot{I}}_{1A}\\ {\dot{I}}_{1B}\\ {\dot{I}}_{1C} \end{array}\right]=Y_{system-Kron}\left(\left[y_{load}\right]\right)\times \left[\begin{array}{c} {\dot{V}}_{1A}\\ {\dot{V}}_{1B}\\ {\dot{V}}_{1C} \end{array}\right]=\left[\begin{array}{c} {\dot{I}}_{1A}\\ {\dot{I}}_{1B}\\ {\dot{I}}_{1C} \end{array}\right]=Y\left(\left[y_{load}\right]\right)\times \left[\begin{array}{c} {\dot{I}}_{1A}\\ {\dot{I}}_{1B}\\ {\dot{I}}_{1C} \end{array}\right]$$

So, defining $h\left(\left[y\right]\right)=\left[\begin{array}{c} {\dot{I}}_{1A}\\ {\dot{I}}_{1B}\\ {\dot{I}}_{1C} \end{array}\right]-Y\left(\left[y\right]\right)\times \left[\begin{array}{c} {\dot{V}}_{1A}\\ {\dot{V}}_{1B}\\ {\dot{V}}_{1C} \end{array}\right],$ a objective function can be achieved this way: $f_{1}\left(\left[y\right]\right)=\max_{1\leq j\leq 3}\left\{|\left[z_{j}\right]-h_{j}\left(\left[y\right]\right)|\right\}$, where j is a coordinate of complex vector.

Other formulations for objective functions are:

$$\left\{ \begin{array}{c} f_{2}\left(y\right)=max_{1\leq j\leq 3}\left(\left|z_{j}-h_{j}\left(y\right)\right|\right)\\ \left[z\right]=\left[\begin{array}{c} {\dot{V}}_{1A}\\ {\dot{V}}_{1B}\\ {\dot{V}}_{1C} \end{array}\right],h\left(y\right)=Y^{-1}\left(y\right).\left[\begin{array}{c} {\dot{I}}_{1A}\\ {\dot{I}}_{1B}\\ {\dot{I}}_{1C} \end{array}\right] \end{array}\right.$$

$$\left\{ \begin{array}{c} f_{3}\left(y\right)=max_{1\leq j\leq 6}\left(\left|z_{j}-h_{j}\left(y\right)\right|\right)\\ \left[z\right]=\left[\begin{array}{c} \begin{array}{c} Re\left({\dot{I}}_{1A}\right)\\ Re\left({\dot{I}}_{1B}\right)\\ Re\left({\dot{I}}_{1C}\right) \end{array}\\ \begin{array}{c} Im\left({\dot{I}}_{1A}\right)\\ Im\left({\dot{I}}_{1B}\right)\\ Im\left({\dot{I}}_{1C}\right) \end{array} \end{array}\right],h\left(y\right)=\left[\begin{array}{c} \begin{array}{c} Re\left({\dot{h}}_{A}\right)\\ Re\left({\dot{h}}_{B}\right)\\ Re\left({\dot{h}}_{C}\right) \end{array}\\ \begin{array}{c} Im\left({\dot{h}}_{A}\right)\\ Im\left({\dot{h}}_{B}\right)\\ Im\left({\dot{h}}_{C}\right) \end{array} \end{array}\right],\left[\begin{array}{c} {\dot{h}}_{A}\\ {\dot{h}}_{A}\\ {\dot{h}}_{A} \end{array}\right]=Y\left(y\right)\times \left[\begin{array}{c} {\dot{V}}_{1A}\\ {\dot{V}}_{1B}\\ {\dot{V}}_{1C} \end{array}\right] \end{array}\right.$$

$$\left\{ \begin{array}{c} f_{4}\left(y\right)=max_{1\leq j\leq 6}\left(\left|z_{j}-h_{j}\left(y\right)\right|\right)\\ \left[z\right]=\left[\begin{array}{c} \begin{array}{c} Re\left({\dot{V}}_{1A}\right)\\ Re\left({\dot{V}}_{1B}\right)\\ Re\left({\dot{V}}_{1C}\right) \end{array}\\ \begin{array}{c} Im\left({\dot{V}}_{1A}\right)\\ Im\left({\dot{V}}_{1B}\right)\\ Im\left({\dot{V}}_{1C}\right) \end{array} \end{array}\right],h\left(y\right)=\left[\begin{array}{c} \begin{array}{c} Re\left({\dot{h}}_{A}\right)\\ Re\left({\dot{h}}_{B}\right)\\ Re\left({\dot{h}}_{C}\right) \end{array}\\ \begin{array}{c} Im\left({\dot{h}}_{A}\right)\\ Im\left({\dot{h}}_{B}\right)\\ Im\left({\dot{h}}_{C}\right) \end{array} \end{array}\right],\left[\begin{array}{c} {\dot{h}}_{A}\\ {\dot{h}}_{A}\\ {\dot{h}}_{A} \end{array}\right]=Y\left(y\right)\times \left[\begin{array}{c} {\dot{I}}_{1A}\\ {\dot{I}}_{1B}\\ {\dot{I}}_{1C} \end{array}\right] \end{array}\right.$$

Thus, load estimation consists of determining the vector [y_load_] that optimizes the objective functions f1, f2, f3 and f4, leading them to assume the lowest value. The different formulations was proposed to verify resolution method's computational performance.

Numerous methods have been proposed for nonlinear function optimization and load estimation [1]. The pattern search method was chosen due to its computational simplicity and robust implementation in MATLAB, as well as the limited number of existing studies utilizing this method for load estimation. It is a derivative-free method as it does not utilize derivatives of the objective function or approximations, and it is a direct search method as it solely relies on point-wise evaluations of the objective function, systematically ordering them.

Given a load vector **y_0_** with n coordinates, the method involves perturbing each coordinate by a factor alpha (a process known as pooling) and evaluating the objective function after each perturbation. The direction $\vec{d}$ that yields the greatest reduction in the objective function value is then explored by selecting the next point $y_{1}=y_{0}+\beta .\vec{d}$ in that direction, where β > α (a process called pattern search). The condition β > α distinguishes pattern search from other methods, as it accelerates the search in the direction that appears to exhibit a pattern of objective function decrease. If pooling again identifies direction $\vec{d}$ as the direction with the greatest reduction in the objective function value, the value of beta is increased, so that $y_{2}=y_{1}+\gamma .\vec{d}$, with γ > β.

In the context of load estimation, [y] is the vector containing the real and imaginary components of the system loads. This vector includes all elements necessary to form the admittance matrices of the loads. Therefore, the proposed objective function for load estimation is constructed by sequentially following these steps:

1. Assembly of the estimated load admittance matrix from vector [y];
2. Addition of these matrices to the nodal admittance matrix of the network y_net_, observing the correct positions, resulting in the estimated system matrix y_system-est_;
3. Application of Kron reduction to the y_system-est_ matrix, obtaining a relationship between the voltages and currents in the feeder;
4. Formulation of the objective function for optimization according to f_1_, f_2_, f_3_, or f_4_;

For the proposed formulation, it is assumed that voltage and current measurements in all three phases of the feeder are available. Additionally, the pattern search optimization method requires an initial estimate, which is formulated for each load by dividing the power measured at the feeder according to the percentage that the nominal power of each load represents in relation to the total installed power, that is:

$$S_{0}^{j}=S_{1}*\frac{S_{nom}^{j}}{\sum_{j}S_{nom}^{j}},\;y_{0}^{j}=\frac{S_{0}^{j}}{V_{nom}^{j}}$$

This calculation method for the initial estimate was chosen due to its straightforward computational implementation and its ability to generate good initial estimates, particularly for the real component of the load.

In an optimization process, constraints can be defined to establish load values that cannot be the result of the estimation, as they imply operating conditions harmful to the system. In the proposed development, it is considered that the loads estimated by the optimization process cannot have a low power factor (the limit was considered to be 0,5), cannot imply high loading for the transformers (maximum limit of 1,25 pu), cannot cause large voltage variations at the buses (must be maintained between 0,95 and 1,05 pu), and, if there is any voltage or current measurement in the system, the calculation with the estimated loads must result in the voltage and current values read by the meter. Mathematically, these constraints are implemented as equations that, after each iteration of the pattern search, calculate the quantities to which the constraint is imposed and increase the value of the objective function if any constraint violation is detected, a process known as penalization. The chosen constraint values reflect the operational conditions that power systems must adhere to in various countries, with the exception of the power factor constraint, which was programmed to be more flexible so as not to render the optimization infeasible.

### 4.2 Implementation description

Thus, to implement the method and discuss results, the simulations was divided in 9 conditions that are explained below:

1. “Impact of the initial estimate”: Evaluation of the initial estimate for each one of the 3 feeders;
2. “Unconstrained objective functions”: Evaluation of 4 unrestricted objective functions for each one of the 3 feeders, comprising 12 estimates;
3. “Power factor constraint”: Evaluation of objective functions f1 to f4 considering restrictions on power factor (pf > 0,5) for each one of the 3 feeders, comprising 12 estimates;
4. “Voltage level constraint”: Evaluation of objective functions f1 to f4 considering restrictions on the magnitude of voltages in the bars (0,93 < *V* < 1,05) for each one of the 3 feeders, comprising 12 estimates;
5. “Constraint based on transformer loading”: Evaluation of objective functions f1 to f4 considering restrictions on transformer loading (< 1,25 pu) for each one of the 3 feeders, comprising 12 estimates;
6. “Constraint based on voltage measurement”: Evaluation of objective functions f1 to f4 considering restrictions on voltage measured in some bars, comprising 22 estimates (many possibilities for positioning the meters are considered);
7. “Constraint based on current measurement”: Evaluation of objective functions formulated based on voltages and currents measured on the feeder and voltages measured on some buses, comprising 12 estimates (In this instance, the processing proved to be time-consuming and computationally expensive, thus limiting the analysis to a single measurement scenario.);
8. “Addition of voltage measurement to the objective function”: Evaluation of objective functions considering restrictions on currents measured in some branches, comprising 34 estimates (many possibilities for positioning the meters are considered);
9. “Addition of current measurement to the objective function”: Evaluation of objective functions formulated based on voltages and currents measured in the feeder and currents measured in some branches, comprising 24 estimates (many possibilities for positioning the meters are considered);

Additionally, the following simplifying assumptions are made:

• All loads are modeled as constant impedances;
• Equipment that can modify the network admittance matrix, such as tap changers and reactive compensators, are considered deactivated;
• The load estimator knows the connection points of each load to the system and the number of admittances required to define each load's admittance matrix.

These simplifications aim to focus the discussion on the application and performance of the optimization process, with the understanding that the aforementioned points can be addressed in future research.

The methodology described in this Section was implemented for simulation using the OpenDSS (Open Distribution System Simulator) as an in-process Component Object Model (COM) server, which is implemented from a Dynamic Link Library (DLL). This process is part of Matlab script. Matlab, a software tool with an extensive library of predefined functions, that enhances the accessibility and efficiency of technical programming tasks (CHAPMAN, 2015).

In summary, MATLAB utilizes the OpenDSS interface to retrieve all circuit data, obtains the network admittance matrix [Y_net_] without loads, executes the load estimation process, and compares the obtained results with the actual loads, calculating individual and statistical error indices.

The Matlab routine supporting the tool comprises scripts in the <.*m*> format, generating files specifically designed for utilization by OpenDSS. The following sections describe the Matlab routine used to generate all the data. This routine is available in the github repository under https://10.5281/zenodo.10731761 [3]. The github details are:

Repository name: l-PROT/LPROT-LoadEstimation

Data identification number: doi:10.5281/zenodo.10731761

Direct URL to data: https://github.com/L-PROT/LPROT-LoadEstimation

Instructions for accessing these data: The data is available in the public domain

### 4.3 Description of the program's files and folders

The program is in the Load_Estimation_PS folder, which contains two subfolders: “01. Matlab” with the files and routines used and generated by Matlab; and “02. OpenDSS” with the OpenDSS files used for the simulation.

The “02. OpenDSS” folder has the following subfolders: “IEEE 13 Barras”; “IEEE 34 Barras”, “IEEE 37 Barras” and “IEEE 123 Barras”. Inside each of these folders are the .dss extension files and others needed for OpenDSS to compile each of these files. Any of these circuits can be opened with the OpenDSS graphical interface and simulate them. These files were copied from the OpenDSS installation folder, specifically from the “IEEETest Cases” subfolder.

Inside the “01. Matlab” folder, there are main files responsible for the program:

• main_program.m: main routine, file to be executed by the user to perform load estimation;
• gera_dados.m: - generate_data.m: routine that simulates the chosen system using the COM interface with OpenDSS and returns system-related data;
• divideSystems: routine that divides the data generated by the "gera_dados.m" routine by measurement area, if chosen by the user;
• opti_ybus.m: function to be optimized for load estimation;
• userData.m: file with the user's input options. It is recommended that only this file be edited by the user;
• matrizes.mat: file with the main data of the simulated system;
• matrizesN.mat: file with the main data of the simulated system related to measurement area N.

In addition to these files, there are subfolders:

• “common”: auxiliary functions that can be used by any routine or function:
  ○ defineYLoad.m – defineYLoad(Yest, Ynet, Yposition): A function that adds the load admittances present in the Yest matrix to the Ynet network admittance matrix, returning the result of the sum. The real matrix Yest has dimensions $n_{l}\times 2$, where n_l_ is the number of loads. The two columns refer to the real and imaginary parts of the admittances. Each row of the matrix contains the real and imaginary parts of one of the load admittances. By converting the admittances to complex form, each admittance is added to the element whose position within the Ynet matrix is defined by the Yposition vector.
  ○ inverseZY.m – inverseZY(vector): receives a matrix vector $\left[a_{ij}\right]$ of dimensions $n_{l}\times 2$ and returns a matrix $\left[b_{ij}\right]$ of the same dimensions, such that:
    $b_{i1}+i.b_{i2}=\frac{1}{\left(a_{i1}+i.a_{i2}\right)}$
  ○ modifyVector.m – modifyVector(vector): receives a matrix vector $\left[a_{ij}\right]$ of dimensions $n_{l}\times 2$ and returns a matrix $\left[b_{ij}\right]$ of dimensions $2n_{l}\times 1$, such that:
    $\left\{ \begin{array}{c} \left[b_{i1}\right]=\left[a_{i1}\right],\;if\;i\leq n_{l}\\ \left[b_{i1}\right]=\left[a_{\left(i-n_{l}\right)2}\right],\;if\;i>n_{l} \end{array}\right.$
  ○ unModifyVector.m – unModifyVector(vector): receives a matrix vector $\left[a_{ij}\right]$ of dimensions $2n_{l}\times 1$ and returns a matrix $\left[b_{ij}\right]$ of dimensions $n_{l}\times 2$, such that:
    $$\left\{ \begin{array}{c} \left[b_{i1}\right]=\left[a_{i1}\right]\\ \left[b_{i2}\right]=\left[a_{\left(i+n_{l}\right)1}\right] \end{array},\;\text{where}\;i\leq n_{l}\right.$$

The other folders, whose functions will be discussed later, are:

• “gera_dados”: contains the functions used by the “gera_dados.m” routine, described in Section 3.3;
• “log”: contains the result files of simulations generated by the “main_program.m” routine;
• “main_program”: contains the functions used by the “main_program.m” routine, described in section 3;
• “opti_ybus”: contains auxiliary functions used to optimize the “opti_ybus.m” function, described in Section 3.5.

The entire process is carried out from the main routine main_program.m. It is what calls the other routines related to the process.

### 4.4 Division of the algorithm into steps

**Fig. 3:**
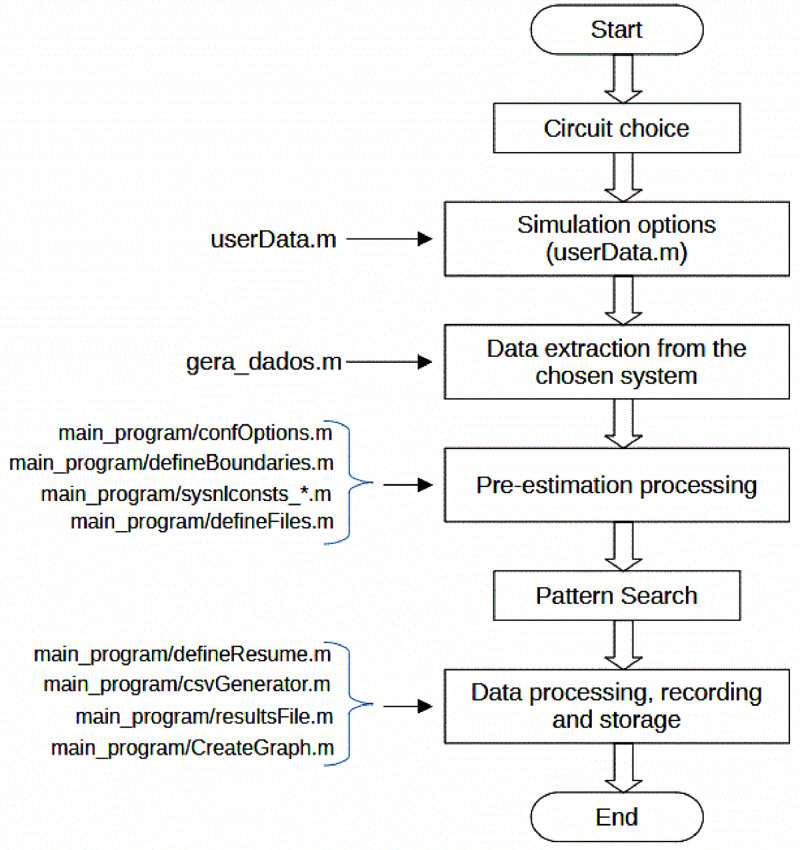
Flowchart of the load estimation process.

The description of this routine will be used to comment on the other routines and functions. The tasks performed by this routine, depicted in Fig. 3, in sequence, are:

1. User selection of the circuit to be simulated;
2. User-defined simulation options through the "userData.m" routine;
3. Executes the “gera_dados.m” routine to obtain data from the chosen system;
4. Processes the data for estimation, which consists of the following substeps:
  a. Processes the system data to obtain the input data for the "opti_ybus.m" function;
  b. Processes the system data to obtain the input data for the "patternsearch" function;
  c. Creates lists for evaluating the estimation, defines the nomenclature and location for saving log files and result record files and carries out the initial filling of these lists and files;
5. Executes the Pattern Search routine in order to optimize the "opti_ybus.m" function;
6. Processes the results and saves them for processing and logging;
7. Finalization.

## 5 Description of Algorithm Steps and Functions

### 5.1 Choice of simulated circuit

It is defined through a prompt in which the user chooses the value of the “escolha” variable. The program offers 4 options:

• 1 corresponds to the IEEE 13-bus circuit;
• 2 corresponds to the IEEE 34-bus circuit;
• 3 corresponds to the IEEE 37-bus circuit;
• 4 corresponds to the IEEE 123-bus circuit.

Any other choice not listed prompts the user again.

### 5.2 Definition of user simulation options - userData.m routine

It occurs when the “userData.m” routine is executed. This routine is the second user interface with the program, in addition to the prompt. It defines the “mainset” structured variable that contains the user's simulation settings. The attributes that the user can modify are: mainset.version: a natural number between 1 and 4 corresponding to f1 to f4 implementation. The relationship between each number and the calculation of the objective function is shown below. In the notation used below, zj is considered to be the j^th^ coordinate of the z vector.

Where Y(y) is the matrix relationship between the three-phase voltages and currents on the feeder obtained from nodal analysis, as a function of the vector y of estimated loads. Any other value for the mainset.version variable works on an experimental basis and does not guarantee the consistency of the results.

**mainset.restriction**: Enables restrictions to be applied to the objective function.

• mainset.restriction=1 enables power factor restriction;
• mainset.restriction=2 enables voltage level factor restriction;
• mainset.restriction=3 enables restriction on transformer loading;
• mainset.restriction=4 enables restriction on voltage measurements taken on busbars;
• mainset.restriction=5 enables a restriction on current measurements made on network elements;
• any other value for this variable runs the optimization function without restriction.

Other experimental fields within the mainset structure, which are not incorporated into the userData.m file, but which can be modified by the user are:

• mainset.const: multiplier constant to increase all the dimensions of the search space (programmed with a constant value equal to 1 so as not to interfere with the operation of the algorithm);
• mainset.dom: modifies the search domain of the optimization function between the impedance (mainset.dom=1) and admittance (mainset.dom=2) options. It was programmed with a value of 1 to maintain the search domain between admittances. The operation of the routine for the impedance option is not guaranteed.
• mainset.method: defines the search method. The program was originally designed to optimize with several different search methods. The methods that have been incorporated so far are optimization using pattern search (mainset.method=0) and genetic algorithms (mainset.method=1). The variable was programmed to use only the pattern search function. The use of GA was incorporated on an experimental basis.

All the repository results were generated without modifying the default value of these last 3 mainset attributes. Modifying them leads to results not described in the repository or unexpected results. The operation of the program is not guaranteed by modifying these variables.

Furthermore, in the userData.m file, the user can modify the value of the variables relating to the busbars that contain voltage and current measurements. These variables are:

• barraVmedx: list of the number of bars in the circuit IEEEx bars that have voltage measurements, where x is the number of bars in the circuit. The name of the bars must be identical to the name of the bar in the native OpenDSS file for the circuit.
• elemImedx: list of network elements in the IEEEx busbar circuit in which current measurement is carried out. The nomenclature of the elements must be the same as the corresponding element modeled in the native OpenDSS file.

### 5.3 Obtaining system data – “gera_dados.m” routine

**Fig. 4:**
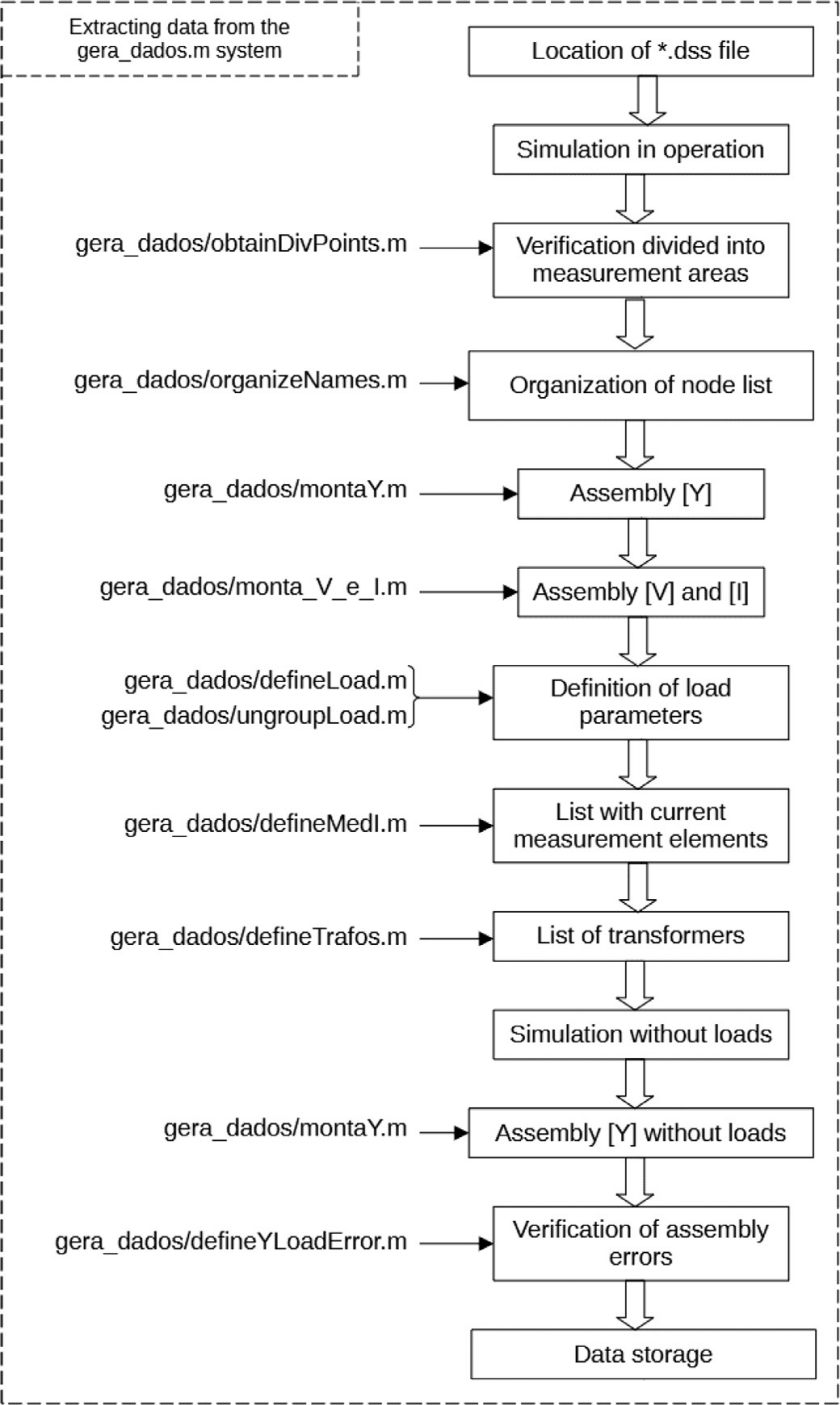
Flowchart of the process carried out by the gera_dados.m routine.

The “generate_data.m” routine, based on the selected circuit for simulation, locates the file in the OpenDSS folder, and uses the OpenDSS COM interface to execute the file corresponding to the circuit. Figure 4 presents the routine steps. Finally, the routine stores the main variables generated in the “matrizes.mat” file, in list form. The purpose of generating data in list form is to make it easier for the user to interpret and debug. The “matrizes.mat” file contains the following variables:

• escolhadiv – variable that indicates whether the user would like to divide the circuit into measurement areas. The division will only be possible if the program identifies in the list of points chosen by the user to contain measurement, any bar with voltage and current measurement other than the feeder bar;
• barraVmed – list of voltage measurement points, imported from the userData.m routine;
• elemImed – elements with current measurement, imported from the userData.m routine;
• barras – variable that indicates the number of bars in the simulated circuit;
• name – string in the form “IEEEx” that indicates the name of the circuit, in which x is the number of busbars;
• divpoints – when the same busbar has voltage and current measurements, it can be used to divide the circuit into two subsystems, as if it were a feeder. The divpoints routine identifies these busbars and adds them to this list;
• subredes – a structure which, if the system is divided into measurement areas, contains lists of the busbars present per subrede;
• node_order – list of circuit busbars with clear identification of feeder busbars, busbars with voltage measurement and busbars with current measurement;
• ptos_medV – number of (single-phase) busbars on which voltage is measured;
• ptos_medSource – number of single-phase busbars on the feeder (usually 3);
• Ysistema_lista – list that contains the admittance matrix of the complete circuit, considering the loads;
• Vorder – list that contains the nodal voltage vector, rows in the same order as the rows in the node_order list;
• Iorder – list that contains the vector of current injections, rows in the same order as the rows in the node_order list;
• Load – list of circuit loads and their electrical attributes;
• Yraiz – vector that contains the true admittance of the loads in the circuit;
• Zraiz – vector that contains the true impedance of the loads in the circuit;
• Sraiz – vector that contains the true power consumed by the loads in the circuit;
• Ypos – list extracted from the 'Load' list with the position of the loads within the Yrede_list matrix, so that adding the admittances of the Yraiz vector in the Yrede_list positions indicated by this list results in the admittance matrix of the circuit with the Ysistema_list loads;
• medi_list: list of elements where current and attributes are measured;
• ptos_medI – number of elements with current measurement;
• trafoList – list of transformers in the system and attributes;
• Yrede_list – list that contains the admittance matrix of the system without the loads;
• Yload_list – list that contains the difference matrix between the matrices contained in Ysistema_list and Yrede_list, i.e., it only considers the system loads;
• YLoadError – real number that symbolizes the error involved in assembling the admittance matrix.

### 5.4 Location of the file to be simulated

The routine uses the variable choice, defined in the main routine, to find out the user's choice. A window opens for the user to indicate where the main_program.m file is located. With this indication, the path to the Load_Estimation folder is stored in the string “pasta”. This way, files related to OpenDSS and auxiliary routines can be located on any computer,^4^ and the functions used by the routine and auxiliary functions can be loaded into the Matlab path.

### 5.5 Simulating the circuit with loads from the COM interface

For each possible choice the user makes, the “caminho1” and “caminho2” variables store the paths to each file in the “02. OpenDSS” folder relating to the simulated circuit. The lists of voltage and current measurement bars are loaded and the number of bars in the circuit is set. The COM interface is used to simulate the circuit without opening the OpenDSS interface in the loaded condition. This interface consists of structures and functions that can be used by Matlab. For example, the DSSText structure is used to give commands involving OpenDSS. Thus, the row DSSText.command = ‘Compile < path and name of the .dss file to be compiled>’ makes OpenDSS compile the specified file. The strings are manipulated to compile the file corresponding to the system the user has chosen. Other commands are used to change the model of all the loads to constant impedance, disable voltage regulators and transformer tap controllers before compilation. After this simulation, other COM interface structures provide all the information of interest about the system, such as the list of busbars, voltage, current and admittance matrices, etc. The structures most commonly used throughout the text are DSSCircuit, which contains general variables relating to the circuit (admittance matrices, nodal voltage vectors, etc.); DSSElement, which contains data relating to the circuit elements and DSSLoads, with data specific to the system loads.

### 5.6 Checking whether the circuit can be divided

After the simulation, the *obtainDivPoints(DSSElement, barraVmed,elemImed)* function compares the lists of voltage measurement points and current measurement elements, identifies the bars with voltage and current measurements, makes a list of the points where the circuit can be divided into smaller subnetworks (measurement areas) and asks the user at the prompt if they prefer to subdivide the system (option 1). The user's choice is stored in the variable escolhadiv. When the user chooses to divide the system, the gera_dados routine proceeds normally, but at the end of the routine, the divideSystems.m program saves the data for each measurement area in a matrizesX.mat file, analogous to the “matrizes.mat” file, where X is the number of the measurement area. All the results presented were designed without using this feature. gera_dados/obtainDivPoints.m - obtainDivPoints(DSSElem, barraV,elemI): function which, from the network elements with current measurement specified in the “elemI” list, obtains the model of these elements in OpenDSS from the DSSElem object, checks whether the terminals of these elements are present in the list of points with voltage measurement “barraV”. If so, it is a busbar with both voltage and current measurement, and therefore able to divide the circuit into subnetworks. Returns a list of busbars in this condition.

### 5.7 Organization of the list of circuit nodes

The list of nodes in the simulated circuit is obtained from the DSSCircuit.YNodeOrder and DSSCircit.AllNodeNames variables in the DSSCircuit structure. The list contains the identification of the circuit nodes, usually numeric format with some alphanumeric characters, with no apparent standardization. From these lists, at this stage, the “node_order” list is generated, which is a list of bars with standardized identification, in which the feeder bars and the bars that contain voltage measurement are marked with the asterisk character, for easy identification, and occupy the first positions in the list.

Thus, the node_order list is organized to follow this order: feeder busbars in the first positions, followed by busbars with voltage measurement and busbars with current measurement. In the last positions are the unmeasured busbars. This order is achieved using matlab's sortrows function, which organizes a list in alphabetical order. The organizeBus(barraVmed, DSSCircuit, DSSElements, DSSSolution, divpoints) function processes the list of bars so that the native “sortrows” command has the expected effect. gera_dados/organizeBus.m – organizeBus (barraV, DSSCirc, DSSElem, DSSSol, divpoint): receives as arguments DSSCirc, DSSEleme and DSSSol objects from the COM interface, the list of division points of the divpoint circuit and the list of bars with voltage measurement barraV and has the purpose of modifying the naming of OpenDSS native bars in order to:

(a) allow simple identification of the feeder, the current and voltage measurement points and the subsystem to which the busbar belongs (in the case of measurement areas);
(b) allow the busbars to be sorted, using the sortrows command, so that the generator busbars are in the first positions and the voltage measurement busbars come next;
(c) when there is more than one measurement area, the busbars must be sorted, from the sortrows command, so that the busbars of measurement area 1 are in the first positions, followed by the busbars of measurement area 2 and so on;
(d) within each measurement area, the members must be sorted according to item 2, with the generator being replaced by the main measurement, which gives rise to the measurement area in question For this purpose, the general naming structure of the lists will be adopted as follows:
  (SS)_(MA/MT)_(Barra).(Fase) or (SS)_(Barra).(Fase)
  where:
  ○ SS = Subsystem, indicates the measurement area in which the busbar is located. If the system is not divided into measurement areas, SS will be equal to 01 for all busbars. The correspondence between each busbar and its measurement area is based on the network's incidence matrix and the fact that it is radial;
  ○ MA/MT = Feeder marking/Voltage measurement marking, indicates whether the busbar belongs to the system feeder/measuring area or whether the busbar has voltage measurement.
    ■ If it is a feeder/generator busbar, MA/MT = ‘***’;
    ■ If it is a busbar with voltage measurement only, MA/MT = ‘*_(tag_ordem)’.
      Ps: (tag_ordem) is a two-digit number to sort the busbars according to the medVODSS list;
    ■ If it is a busbar with voltage measurement and there is a current measuring element such that the current is incident on that busbar, this could be a point of division of the circuit into measurement areas. In this case, MA/MT = '**_(tag_ordem)'. Ps: (tag_ordem) is a two-digit number to sort the busbars according to their order in the medVODSS list;
    ■ If the busbar is neither a feeder busbar nor has voltage measurement, this marker will be absent in the nomenclature, MA/MT = '', the busbar nomenclature will be of the type (SS)_(Barra).(Fase).
      ✓ (Barra) - busbar identifier, as provided by OpenDSS, corrected by a naming system described in organizeNames.m;
      ✓ (Fase) - 1 and/or 2 and/or 3, depending on which phase of the busbar will be used. When more than one phase is used, the phases are separated by dots (e.g. 00670.1.2 refers to phases 1 and 2 of busbar 00670).

Since the identification of the buses is numeric, the organizeNames.m function is used within the organizebus.m routine to standardize the naming of the buses. gera_dados/organizeNames.m – organizeNames(name): receives a list of busbar names and modifies it so that each busbar is associated with a 5-character identifier, with missing characters being filled in with zeros. When the busbars are two-phase or single-phase, the busbar names discriminate the phases using dots (e.g. “23.1.2” means busbar 23 is connected to the circuit on phases A and B only). These phase indicators are maintained by the function and do not count as bar nomenclature.

In addition to the modified YNodeOrder list of busbars, the organizebus.m function returns the divpoint corrected list of circuit division points, if there are points that cannot be in different subnets; it also returns a “subredes” list of busbars belonging to each subnet, if applicable. The YNodeOrder list is submitted to matlab's sortrows function, resulting in the node_order variable.

### 5.8 Assembly of the nodal admittance matrix

The node_order variable is used to count how many single-phase nodes refer to feeders and how many refer to busbars with voltage measurements. After that, the montaY(DSSCircuit, YNodeOrder, Ysistema_list, escolhadiv) function retrieves the elements of the admittance matrix of the COM interface system from the DSSCircuit.SystemY attribute and processes them to return the Ysistema_ list. gera_dados/montaY.m – montaY(DSSCirc, nos, nos_ordem): a function that, from the OpenDSS DSSCirc (DSSCircuit) object, retrieves the admittance matrix of the network elements, whose rows and columns follow the same order as the “nos” bar list. The function reorders the matrix to follow the same order as the list of bars “nos_ordem” and generates a list containing the admittance matrix, in which the rows and columns are identified with the names of the respective nodes.

### 5.9 Assembly of nodal voltage and injected current vectors

The attributes DSSCircuit.YNodeVarray and DSSCirc.YCurrents contain the elements of the vectors of nodal voltages and injected currents, respectively. The function monta_V_e_I(DSSCircuit, YNodeOrder, Ysistema_list, escolhadiv) retrieves these elements and processes them to obtain the Vorder and Iorder lists.

gera_dados/monta_V_e_I.m – monta_V_e_I(DSSCirc, nos, Ysis_list,choice): obtains the nodal voltage and injected current vectors from the DSSCirc object of the COM interface, whose ordering is the same as the “nós” list and processes them to generate the Vorder and Iorder lists with bus voltage values and injected currents. In the Vorder list, the columns are identified as follows:

• Column 1: identification of the nodes, according to node_order;
• Column 2: value of the complex injected voltage at each node;
• Column 3: value of the voltage modulus in pu at each node;
• Column 4: vector corresponding to the following operation between matrices contained in the Ysistema_list and Iorder lists: ${\left[Y_{sistema_{list}}\right]}^{-1}*\left[I_{order}\right]$
• Modulus of the difference between the values in column 4 and column 2, in order to check for possible processing errors.

The Iorder list is similar, except that it does not contain the column for the modulus of the quantity in pu.

### 5.10 Definition of the list of circuit loads and positions in the admittance matrix

The defineLoad(DSSCircuit, DSSElement, DSSCktElement, DSSLoads, node_order, barraVmed, barras, escolhadiv) function extracts the load list from the DSSLoads structure and returns the “Load” load list and the “verify” variable, which is an error indicator when assembling this list.

To make it easier for the optimization algorithm to work, the three-phase loads in the load list are broken down into single-phase loads using the ungroupLoad(Load,Vorder) function.

gera_dados/defineLoad.m – defineLoad(DSSCirc, DSSElem, DSSCktElem, DSSCarga, nos_ordem, busmedV, buses, choice): from the objects DSSCirc, DSSElem, DSSCktElem, DSSCarga of the COM interface, the list of nodes nos_ordem, the list of busbars with voltage measurement busVmed, the number of busbars of the circuit buses and the choice escolha if the circuit is subdivided into measurement areas, it returns a list of loads consisting of a table in which each row contains the attributes of one of the loads in the following order:

Subsystem: indicates the measurement area in which the load is located;

Name: name of the busbar and phases to which the load connects, obtained from the DSSElem.BusNames object and the DSSCarga.Name element;

Tensão (V): nominal voltage of the load, obtained from the variable DSSCarga.kV;

W: nominal active power of the load, obtained from DSSLoad.kW;

Var: nominal reactive power of the load, obtained from DSSCarga.kvar;

Conn: load connection to the system, which can be Phase-Earth, Phase-Phase, 3Ph or Delta. This data is obtained by processing the information from the nodes where each load connects.

Bus: nodes to which the load connects, obtained from the node_order list, with standardized nomenclature;

Ypos: row and column indices that demarcate the position within the nodal admittance matrix where the admittance of that load is located;

Yprim: admittance matrix of the load as a network element;

Ycarga: main admittance representing the load;

P1(kW): real active power consumed by the load, obtained from the DSSElem.Powers object;

Q1(kW): real reactive power consumed by the load, obtained from the DSSElem.Powers object;

The real number verify is obtained from the Yprim column by summing the rows of the matrix. It is known that this number must be close to zero, which is an indication of errors in the algorithm for assembling and processing this matrix.

gera_dados/ungroupLoad.m - ungroupLoad(Load, Vorder): transforms the Load load list so that all loads have only a phase-to-phase or phase-to-ground connection. Thus, star loads are broken down into 3 phase-to-earth loads while delta loads are broken down into 3 phase-to-phase loads. The Vorder nodal voltage list is used for this and the decomposed load list is returned.

### 5.11 Generation of a list with the elements in which current is measured

This list will be used to build constraints or to improve the objective function. This is done using the defineMedI(DSSCircuit, DSSElement, Vorder, barraVmed, elemImed, node_order, barras, escolhadiv) function. It should be noted that the system feeder is always in this list, and is always the first element.

\gera_dados\defineMedI.m – defineMedI(DSSCirc, DSSElem, Vordem, barraV_med, elem_Imed, no_ordem, buses, choice): uses the OpenDSS objects DSSCirc and DSSElem, the list of current measurement elements elem_Imed, the list of voltages in the Vordem nodes, the list of voltage measurement bars barV_med, the list of nodes no_ordem, the number of buses and the user's choice of dividing the circuit into measurement areas choice. It returns a list in table form of current measuring elements, the columns of which are in the following order:

‘Elemento’: name of the network element, obtained from the DSSCirc object;
Yprim: admittance matrix of the element's network elements, obtained from the DSSElem.Yprim variable;
Node1: busbar 1 to which the network element is connected;
Subsystem1: measurement area in which busbar 1 is located;

Indice Node 1: position in the no_ordem list where busbar 1 is located. If the busbar contains more than one phase, this field will contain a column matrix with more than one number, each indicating the position of a phase;

V1: nodal voltage in each phase of busbar 1, obtained from the Vorder list;
I1: The current injected into the network element from busbar 1, obtained from the DSSElem.Currents variable;
S1: Power flow through the network element from bar 1, obtained from the DSSElem.Powers variable;
Node2: busbar 2 to which the network element is connected;
Subsystem2: measurement area in which busbar 2 is located;
Indice Node 2: position in the no_ordem list where busbar 2 is located, analogous to the Index Node 1 field.
V2: nodal voltage in each phase of busbar 2, obtained from the Vorder list;
I2: The current injected into the network element from busbar 2, obtained from the DSSElem.Currents variable;
S2: Power flow through the network element from busbar 2, obtained from the DSSElem.Powers variable.
Imed: current symbolizing the value obtained by the current meter, numerically equivalent to column 13.

### 5.12 Generation of a list of transformers in the circuit

This list will be used to build constraints based on the loading of the transformers. The list is built by the defineTrafos(DSSCircuit, DSSElement, node_order, barras, escolhadiv) function.

gera_dados/defineTrafos.m – defineTrafos(DSSCirc, DSSElem, no_ordem, buses, choice): extracts the information on the system's transformers from the DSSCirc.Transformers structure and information from the DSSElem structure and builds a list of the circuit's transformers, with the following attributes:

Nome: name of the transformer obtained from the DSSCirc.Transformers.Name variable.
Kva: apparent nominal power of the transformer, obtained from the variable DSSCircuit.Transformers.kva.
Yprim: admittance matrix of the transformer, obtained from DSSElem.Yprim.
Bus1: name of bus 1 to which the transformer is connected;
Indice Bus1: index of the positions of the nodes defined in the "Bus1″ field in the no_ordem list;
Subsystem: área de medição em que a barra 1 está localizada;
Bus2: nome da barra 2 na qual o transformador está documentado;
Indice Bus2: índice das posições dos nós definidos no campo “Bus2” na lista no_ordem;
Subsystem: measurement area in which busbar 1 is located;

### 5.13 Simulating the circuit without loads from the COM interface

Similarly to item b, the circuit is simulated again, but now using the command line “DSSText.command = ‘batchedit Load..* enabled=no’” ”, which disables the loads. Then, the list Yrede_list that contains the matrix of admittance without the charges is obtained from the montaY function in the same way as in item e.

### 5.14 Checking assembly errors in the matrix with loads

The defineYLoadError(Ysistema_list, Yrede_list, Load, node_order, verify) function assembles the load admittance matrix in two different ways and returns the system admittance matrix considering only the Yload_list loads and the YLoadError error index.

\gera_dados\defineYLoadError.m – defineYLoadError(Ysis_list, Ynet_list, Carga, no_ordem, verifica): obtains the Ycarga_list matrix in two different ways:

• From the Load load list, summing the admittances of the loads in their respective positions (Ypos column) in a null matrix. At the end, the elements of this matrix are summed and the sum is stored in the variable verifica2;
• A partir da subtração $\left[Y_{sistema_{list}}\right]-\left[Y_{rede_{list}}\right]$ of the admittance matrix with the loads and the admittance matrix with the loads. At the end, the elements of the matrix are summed and the sum is stored in the variable verifica3.

At the end of the process, the differences between the variables verify, verify2 and verify3 are taken, of which the largest module is returned to the user in the form of an error index (YLoadError).

### 5.15 Storage of the main variables to be reused by other routines

The variables are stored in the matrizes.mat file and a warning “Dados principais gerados” (Main data generated) is issued to the user. If the user has chosen to subdivide the system into measurement areas, the divideSystems.m routine is triggered. As the results obtained were not based on this routine, it will not be described.

### 5.16 Processing of system data according to the function opti_ybus.m

At the end of the gera_dados.m routine, if the user has chosen to divide the circuit into measurement areas, they must choose at a prompt which of these areas should be used for simulation. The measurement areas are stored in the structured variable subredes, and the ordering of the lists in this variable defines the number of each subrede and also defines the matrizesX.mat file in which the specific data for that subrede is saved. Thus, once the user has chosen the number of the subnet to be simulated, the matrizesX.mat file is loaded. The data from the file corresponding to the simulated system (matrizes.mat or matrizesX.mat) is loaded into matlab and stored in the structured variable “dados”. Thus, all the variables described in Section 3.3 become attributes of the “dados” structure.

Processing consists of accessing the lists in the “dados” structure and making them available in numerical format, to be used as arguments for the “opti_ybus.m” function and other processing functions. The post-processed data in this way is stored in the “circuit” structure. The fields in this structure are:

• circuit.Vmed: vector with actual voltage on the busbars;
• circuit.Vpu: vector with the modulus of the voltage on the busbars in pu;
• circuit.Iinj: vector of nodal current injections;
• circuit.Imedido: list of elements in which current is measured;
• circuit.Ssource: apparent power measured at the feeder;
• circuit.Yrede: admittance matrix of the chosen circuit without the loads;
• circuit.Ysistema: admittance matrix of the chosen circuit with the actual loads;
• circuit.Yload: difference between the circuit.Ysistema and circuit.Yrede matrices;
• circuit.Yprim: list with the admittance matrix of the elements in which current is measured and the index of the position of the busbars in which these elements are connected within the busbar voltage vector;
• circuit.Ypos: list with the position of each load admittance within the nodal admittance matrix;
• circuit.ptos_medSource: number of single-phase nodes on the feeder;
• circuit.ptos_medV: number of single-phase busbars where voltage is measured;

Except for the fields Vpu, Ssource and Yload, the other fields in this structure will be passed as arguments to the opti_ybus.m. function. These three fields will be arguments to other data processing functions.

## 6 Pre-Estimation Processing

### 6.1 Processing of system data according to the patternsearch function

At this stage, most of the fields in the search structure, which contains the parameters for the search, are filled in.

Initially, the vectors dados.Yraiz, dados.Zraiz and dados.Sraiz are processed, which contain, respectively, the admittances, impedances and real powers of the loads and store them in the search structure (variáveis search.Yraiz, search.Zraiz and search.Sraiz), which contains the parameters for optimization. The other fields of the search variable filled in at this stage are those that will be used as arguments for the patternsearch function^5^:

• search.options: contains the search options characteristic of the patternsearch function. These options are available in the function's tutorial on the Internet^6^;
• search.chute_inicial: vector with the initial load estimate, from which the algorithm is started;
• search.UB and search.LB: search boundaries. These are, respectively, the maximum and minimum values that each admittance component (real part and imaginary part) can take;
• search.nonlcon: pointer to the function representing the non-linear constraints.^7^ If there is no non-linear constraint, this field is left empty.

The options variable is configured from the confOptions(mainset.method, search) function. It defines the search options such as the maximum number of iterations, stopping criteria, size of the search mesh, etc. The vector with the initial estimate is returned by the function defineChuteInicial(dados.Load, circuit.Ssource, mainset.dom) and the vector with the search limits is returned by the function defineBoundaries(search.chute_inicial, search.Raiz, mainset.dom). In the case of constraints, basically all constraints are non-linear. In this case, the patternsearch argument must be a pointer to a function that checks whether these constraints are met. This pointer, depending on the value of the mainset.restriction variable, is stored in the search.nonlcon variable. All the functions that represent non-linear constraints are in the “opti_ybus” folder. They all have the same set of arguments.

• mainset.restriction=1 corresponds to the pointer to the function sysnlconsts_fp(x, circuit.Vmed, dados.medI_list, circuit.Vpu, circuit.Iinj, circuit.Yrede, dados.Ypos, circuit.Y_prim, dados.barraVmed, dados.ptos_medI, dados.ptos_medV, dados.trafoList, mainset.dom);
• mainset.restriction=2 corresponds to the pointer to the function sysnlconsts_magV(x, circuit.Vmed, dados.medI_list, circuit.Vpu, circuit.Iinj, circuit.Yrede, dados.Ypos, circuit.Y_prim, dados.barraVmed, dados.ptos_medI, dados.ptos_medV, dados.trafoList, mainset.dom);
• mainset.restriction=3 corresponds to the pointer to the function sysnlconsts_trafo(x, circuit.Vmed, dados.medI_list, circuit.Vpu, circuit.Iinj, circuit.Yrede, dados.Ypos, circuit.Y_prim, dados.barraVmed, dados.ptos_medI, dados.ptos_medV, dados.trafoList, mainset.dom);
• mainset.restriction=4 corresponds to the pointer to the function sysnlconsts_V(x, circuit.Vmed, dados.medI_list, circuit.Vpu, circuit.Iinj, circuit.Yrede, dados.Ypos, circuit.Y_prim, dados.barraVmed, dados.ptos_medI, dados.ptos_medV, dados.trafoList, mainset.dom);
• mainset.restriction=5 corresponds to the pointer to the function sysnlconsts_currents(x, circuit.Vmed, dados.medI_list, circuit.Vpu, circuit.Iinj, circuit.Yrede, dados.Ypos, circuit.Y_prim, dados.barraVmed, dados.ptos_medI, dados.ptos_medV, dados.trafoList, mainset.dom);

main_program/confOptions.m – confOptions(metodo, busca): receives the search method and the structure with search parameters and configures these structures with the search parameters and options used.

main_program/defineBoundaries.m – defineBoundaries(initial_guess, Root, domain): receives the vector with the initial estimate initial_guess and the vector with the actual admittance values Root and, according to the chosen search domain, returns two vectors and a string. The two vectors are the maximum and minimum search limits and the string verifies that the region between the two vectors comprises the initial estimate.

opti_ybus/sysnlconsts_<restriction>.m - sysnlconsts_<restriction>(x, Vmed, Imedlist, Vp, Inodes, Ynet, Yposition, Yprimaria, nomeVmed, n1, n2, trafList, dominio): general form of the functions in the opti_ybus folder that indicate some linear restriction to be observed. All functions use the same arguments: vector x of admittance of the circuit loads, vector Vmed of nodal voltages, list Imedlist of circuit elements with current measurement, vector Vp of modulus of nodal voltages in pu, vector Inodes of currents injected into the nodes, matrix Ynet of nodal admittance of the circuit without the loads, list Yposition with the positions of all the loads in the matrix of nodal admittances, list Yprimaria with admittance matrices of the network elements in which there is current measurement; list nomeVmed with busbars where voltage is measured, number n1 of busbars with current measurement on an adjacent element and number n2 of busbars where voltage is measured, list trafList of circuit transformers and search domain. The 〈restriction〉 field can take on the values:

• fp: power factor. In this case, for each of the admittances in the x vector, the power factor is calculated and a vector with the difference between each power factor and 0.5 is returned;
• magV: voltage magnitude. In this case, for each of the admittances of x vector, the voltage modulus at each node is calculated in pu and a vector with the difference between 1.05 and each voltage modulus at each node in pu is returned, also returning in the same vector the difference between each voltage modulus in pu at each node and 0.95;
• trafo: transformer loading. In this case, from the list of admittances, the power of each transformer in the trafoList is calculated in pu, and a vector with the difference between 1.25 and this power factor is returned;
• V: voltage measurement. In this case, from the list of admittances, the voltage is calculated at each of the nodes where it is measured. A vector with the difference between the measured and calculated voltages is returned;
• current: current measurement. In this case, from the list of admittances, the current in each network element where current is measured is calculated using its network element admittance matrix. A vector with the difference between the measured and calculated currents is returned.
• *Definition and population of files to store results*

### 6.2 Definition of the name and structure of the output files

Furthermore, at this stage, the nomenclature and location of the files populated with the results are defined using the defineFiles(mainset, dados, search) The output files are stored in a subfolder of the “log” folder, whose name structure is given as follows:

IEEE<NOME>_<DOMINIO>_<HORARIO>_*v*<VERSION>_mv<MV>_mI<MI>_<NC>

Where:

• <NOME>: Identifier of the simulated circuit, usually the number of bars. When it is a subsystem, it will be indicated by the number of busbars in the larger circuit plus the subsystem number. For example, IEEE13_1 is subsystem 1 of the IEEE 13-bus circuit.
• <DOMINIO>: Domain in which the Pattern Search was executed. Y for admittances and Z for impedances.
• <HORÁRIO>: String with the time at which the simulation was carried out. The string format is <ANO><MÊS><DIA><HORA><MINUTO>.
• <VERSION>: Which objective function is being run.
• <MV>: Number of points with voltage measurement, counting the phases. A three-phase bar adds 3 points with voltage measurement.
• <MI>: number of points with current measurement, counting the phases.
• <NC>: indicates whether the simulation was carried out using non-linear constraints. The use of these constraints is marked with the string 'NC'. If the file name does not contain this string, non-linear constraints do not apply.

The results folder contains the subfolders ``Results S'', ``Results Y'' and ``Results Z'', along with the files ``Log.txt'' and ``variables.mat'', store the results for the same test, but in the formats of apparent power of the loads, admittance of the loads and impedance of the loads, respectively. main_program/defineFiles.m - defineFiles(confprinc, data, busca): uses the main configuration structure confprinc, the structure with circuit data and the structure with search data search to return structured variables whose attributes are the paths and names of the files in which the results will be stored. The outputs of this function consist of four structured variables, the fields of one of which, the files^8^. variable, are described below:

• files.folder - path and name of the results folder, within the ‘log’ folder. The folder name follows the previously explained structure;
• files.nomearqlog - path and name of the ‘Log.txt’ file containing the main data and results of the simulation in text mode. This file is inside the results folder;
• files.nomearqmat - name of the ‘.mat’ file that contains the matlab variables used and generated by the simulation, for further processing if necessary. This file is also in the results folder and is called “variables.mat”.

The other 3 structured variables filesS (arqS), filesY (arqY) and filesZ (arqZ) contain analogous fields and are used to contain the name and path of the result recording files, respectively, in the form of power, admittance and impedance. The fields for these variables, indicated using the “files*” notation, are described below:

• files*.nomearqfig - name of the ‘.fig’ file containing the histogram generated by the simulation;
• files*.nomearqresume - name of the '.csv' file with the summary of the load estimation carried out, containing the results and individual errors per bar;
• files*.nomearqtabFreq - name of the '.csv' file containing the frequency table for the errors of the load estimation performed;
• files*.compare1 - name of the '.csv' file containing the comparison between the errors of the estimate made and the errors relating to the initial guess;
• files*.compare2 - same as arq().compare1, but using statistical figures of merit, such as maximum error, average error and standard deviation. These figures of merit are discussed later.

### 6.3 Creation and initial population of the log file

Once the nomenclature and path of the saved files have been defined, the log files are created and populated with initial data using the function initFile(files.arqlog, mainset.version, mainset.const, dados.ptos_medSource, dados.ptos_medV, dados.node_order, dados.barraVmed, dados.elemImed, options, search.LB, search.UB, search.chute_inicial, dados.barras, search.verifyBounds, mainset.method). This function records all these data in the log file in the form of a header.

main_program/initFile.m – initFile(arqlg, ver, con, ptosS, ptosV, nos, barraV, elemI, opt, lb, ub,

init_guess, bus, verificaB, method): creates a file from the path and name to the .txt log file of the arqlg algorithm and writes in it: the ver version of the objective function that to be evaluated; the con constant for expanding the domain of the objective function; the ptosS number of measurement points on the feeder, counting the phases; the ptosV list of network elements that contain current measurement, excluding the feeder; the nos list with busbars sorted according to organize_bus; the barraV list of busbars on which there is voltage measurement; the elemI list with elements in which there is current measurement; the opt structure with the Pattern Search configuration options; the lb lower limit for the estimation algorithm; the ub upper limit for the estimation algorithm; the init_guess initial guess for the estimation algorithm; the bus number of three-phase busbars in the circuit; the verificaB indicating whether the correct value is between the roots; and the method search method used (Fig. 2, Fig. 3, Fig. 4).

### 6.4 Execution of the pattern search function

At this stage, the patternsearch method is executed, preceded by some preparations.

The sub-steps involved in this stage are:

1. Calculation of the function to be optimized at two points: the point of initial estimation and the point with the real admittances (which supposedly should lead to a null function value). The aim of this sub-step is to provide input for subsequent evaluation of the optimization. Once these two values have been calculated, they are stored in the search.finic and search.fmin variables and written to the log files.
2. Recording the system time before applying the function in the search.inicio variable.
3. Application of patternsearch aiming to optimize the opti_ybus.m function. Initially, the arguments of the opti_ybus function are an admittance vector (independent variable) and the following variables are assumed to be constant during the optimization process: the fields Vmed, Imedido, Iinj, Yrede, Ysistema, Ypos, and Y_prim of the circuit structure; the fields ptos_medSource and ptos_medV of the data structure; and the fields const, version, and dom of the mainset structure. Thus, for each independent variable (admittance vector), the opti_ybus function uses circuit parameters to calculate the nodal voltage of the feeder and evaluate its difference from the measured voltage, generating a real number proportional to this difference. The patternsearch optimization function tests various admittance vectors following a predefined order so that the real number generated is zero, which occurs when the admittance vector is equal to the actual admittance vector of the circuit (search.Yraiz).
4. Recording the system time at the end of the simulation in the search.fim variable;
5. Determination of the execution time from the time difference between the search.fim and search.inicio variables;
6. Recording the estimated admittance value in the resultsY.Estimado variables and converting the vector to impedance and power values, and recording them in the respective resultsZ.Estimado and resultsS.Estimado variables. The conversion to impedance values is done by taking the inverse of the estimated admittances (from the inverseZY.m function) and the conversion to power values is done using the defineS.m function, which takes as arguments the list of loads (dados.Load), the vector of nodal currents (circuit.Iinj), the admittance matrix of the network without the loads (circuit.Yrede) and the estimated admittance vector (resultsY.Estimado). The function of the resultsY, resultsZ and resultsS variables in storing the estimation results is discussed in more detail in the next section.
7. Checking whether the solution found for the optimization process violates any of the established restrictions: applying the search.nonlcon pointer to the solution found and checking, from the output, whether the value found violates any restrictions^9^;

### 6.5 Processing, recording and storing results

Population of the results* structure (* = *Y*, Z or S)

The resume structure is populated from the defineResume function. The purpose of this structure is to store the lists that contain the test results. The fields/lists of this structure described for variable S (resultsS) are:

• id – identifier of the magnitude characterizing the result. The options are S, Y, and Z, referring to apparent power, admittance, and impedance, respectively;
• chute_inicial – identical to the search.chute_inicial variable but in matrix format with two columns, the first containing the real part and the second containing the imaginary part;
• Estimado – result of estimating loads in two columns (real part and imaginary part);
• Resume – intended to contain the estimation results and estimation errors for each load. It is generated as a table to make it easier for the user to consult. The resultsS.resume list contains the following fields (for the resultsY.resume and resultsZ.resume lists the fields are analogous):
  ○ Name : identification of the busbar to which the load connects;
  ○ Re(Sraiz): Real part of the load's true power;
  ○ Im(Sraiz): Imaginary part of the load's true power;
  ○ Re(Sest): Real part of the load's estimated power;
  ○ Im(Sest): Imaginary part of the load's estimated power;
  ○ Erro – Abs(Delta): Percentage of the modulus of the difference between the true and estimated powers in relation to the true power.
    $$Erro\;Abs\left(Delta\right)\left(\%\right)=100.\frac{\left|S_{raiz}-S_{est.}\right|}{\left|S_{raiz}\right|}$$

○ Erro – Arg(Delta): Percentage of the difference between the arguments in relation to the true argument.

$$Erro\;Delta\left(Arg\right)\left(\%\right)=100.\frac{\arg\left(S_{raiz}\right)-\arg\left(S_{est}\right)}{\arg\left(S_{raiz}\right)}$$

○ Erro - Real: Percentage error in estimating the real part in relation to the true nominal active power.

$$Erro\;Real\left(\%\right)=100.\frac{Re\left(S_{raiz}\right)-Re\left(S_{est}\right)}{Re\left(S_{raiz}\right)}$$

○ Var9: Percentage of imaginary estimation error in relation to the true nominal reactive power.

$$Erro\;Imag\left(\%\right)=100.\frac{Im\left(S_{raiz}\right)-Im\left(S_{est}\right)}{Im\left(S_{raiz}\right)}$$

• maxResume: contains statistical indices for each of the errors described above. Each of these errors is represented in a column of the list. The row indices of the list related to each of the estimation errors are:
  ○ MaxErro – maximum error among all circuit estimates in VA,^10^ considering only the numerator of the error formulas presented, without multiplying by 100;
  ○ MaxErroPercent – maximum percentage error among all estimates;
  ○ MinErro - minimum error among all circuit estimates in VA,^11^ considering only the numerator of the error formulas presented, without multiplying by 100;
  ○ MinErroPercent – minimum percentage error among all estimates;
  ○ MeanAvgError – average error, arithmetic mean between error modules;
  ○ MeanSysErrorPercent – systematic percentage error, percentage of the sum of the modules of the errors relative to the sum of the true dissipated powers;
  ○ stdAvgError – standard deviation associated with the MeanAvgError error;
  ○ cvErrorPercent – coefficient of variation associated with the average error;
  ○ stdSysError – standard deviation associated with the systemic percentage error;
  ○ mape – mean absolute percentage error, average of the modules of the percentage errors;
  ○ stdMape – standard deviation associated with the mape;
• tabFreq – frequency table associated with the estimation. The rows correspond to the estimation error bands, with 10 equally wide ranges between errors from 0 to 1 %, 9 bands between errors from 1 to 10 %, 9 bands between errors of 10 and 100 %, and one band for errors greater than 100 %. The columns correspond to the cumulative frequency of loads (percentage of loads in relation to the total) whose estimation error does not exceed the index determined by the row;
• hist. – stores histogram data built from the tabFreq list;
• resumeinic e maxResumeinic – analogous to the resume and maxResume lists, but does not use the estimation data for calculation but the initial estimate data, contained in resultsS.chute_inicial;
• compare1 – similar to the resume list, it contains, for each error field defined, the difference between the error modules obtained with the estimation performed and with the initial estimate, in order to show the evolution of the estimation;
• compare2 – similar to the maxResume list, it contains, for each defined statistical index, the difference between the modules of the index obtained with the performed estimation and the index obtained with the initial estimate, showing the evolution of the estimation.

The lists for the S variable are filled in using the defineResume(resultsS,dados) command and the other lists are filled in the same way. main_program/defineResume.m – defineResume(resX, data): from the resX structure already containing the estimation results in terms of the quantity X, it fills in the other attributes of the resX variable as explained above.

### 6.6 Storage of lists from the results* structure in .csv files

Next, the csvGenerator function saves some of the internal tables of the results* structure in .csv files, as these are formats that can easily be imported by other applications. The function is used for each of the magnitudes used to represent the result: S, Y and Z. For magnitude S, the command is csvGenerator(resultsS, filesS) and for the other magnitudes, the command is analogous.

The log file is populated with the results. In addition to containing the results of the tables in the resume* structure, this file also contains simulation-specific results such as convergence time, number of iterations performed, and the optimized value of the function achieved by the algorithm. Much of the data is written by the function resultsFile(resultsY, resultsZ, resultsS, search.tempo, files.arqlog)

main_program\csvGenerator(resX, arqX): The function receives as arguments the resX structure and the arqX structure, which must refer to the same quantities used to express the estimation (e.g. resultsY can only be used together with filesY as arguments to the function). The function then generates the 4 .csv files explained in the definition of the “files*” structure.

main_program\resultsFile(resY, resZ, resS, time, arq): takes as arguments the three results structures in the form res*, the convergence time and the name of the log file arq. It writes the test results to the log file and to the standard matlab output screen.

### 6.7 Histogram generation

The CreateGraph(resultsS, filesS) function is used to generate a histogram of the power results. For the other quantities, similar function calls are made.

main_program/CreateGraph.m – CreateGraph(resX, arqX): receives as arguments the resX structure and the corresponding nomenclature of the histogram file defined in arqX and generates a .png file with the histogram of the estimate, as well as displaying the histogram from matlab's standard output. The .png file is saved according to the path stored in the arqX structure.

### 6.8 Recording variables in .mat file

The main structures generated are stored in a file for further processing.

## 7 Data Generation

Data were acquired using computer simulation with Matlab 2015a and OpenDSS 9.3.0.2 software. The computer had the characteristics:

Processor: Intel(R) Core(TM) i7-8700 CPU @ 3.2 GHz 3.19 GHz

RAM: 8GB

Operating System: Windows 10 Education 64 bits

## Limitations

The limitation of the data lies in the utilization of IEEE test feeders rather than actual existing real-world feeders.

## Ethics Statement

The authors have read and follow the ethical requirements for publication in Data in Brief and confirm that the current work does not involve human subjects, animal experiments, or any data collected from social media platforms.

## CRediT authorship contribution statement

**Leonardo Ramos Pereira:** Conceptualization, Methodology, Software, Validation, Formal analysis, Data curation, Writing – original draft, Writing – review & editing, Visualization. **Giovanni Manassero:** Conceptualization, Methodology, Software, Validation, Formal analysis, Data curation, Writing – original draft, Writing – review & editing, Visualization, Funding acquisition.

## Data Availability

The performance of the pattern search direct search method in solving load estimation problems (Original data) (Mendeley Data)

## Footnotes

1 In the original repository tables, the number of significant figures is high. In this article, we chose to present the tables with two decimal places to save space and preserve readability.

6 https://www.mathworks.com/help/gads/pattern-search-options.html

7 To learn how to configure nonlinear constraints for the patternsearch function, visit: https://www.mathworks.com/help/gads/description-of-nonlinear-constraint-solver.html

9 To understand the type of response of a function used as a non-linear constraint, visit: https://www.mathworks.com/help/gads/description-of-nonlinear-constraint-solver.html

10 Error in VA because the resultsS structure is being used as an example. If it were the resultsZ structure, the error would be in ohms.

11 Error in VA because the resultsS structure is being used as an example. If it were the resultsZ structure, the error would be in ohms.

## Footnotes

2 In the original repository tables, the number of significant figures is high. In this article, we chose to present the tables with two decimal places to save space and preserve readability.

3 In the original repository tables, the number of significant figures is high. In this article, we chose to present the tables with two decimal places to save space and preserve readability.

4 As long as the internal structure of the Load_Estimation folder is kept the way it was presented.

5 Although the example used for the search method is pattern search, the same goes for any other search method, the option mainset.methd = 1, which uses GA (genetic algorithm) also requires these same parameters.

8 The name ``files'' is changed to ``arq'' in the function definition. The names ``filesS'', ``filesY'' and ``filesZ'' are changed to ``arqS'', ``arqY'' and ``arqZ'' respectively.
